# Efficiency evaluation of a lab-scale photoelectric precipitator for particulate matter emission reduction

**DOI:** 10.1007/s40201-024-00913-1

**Published:** 2024-07-16

**Authors:** Kiarash Abdollahzadeh, Somayeh Soleimani-Alyar, Rasoul Yarahmadi

**Affiliations:** Air Pollution Research Center, Department of Occupational Health Engineering, Iran University of Medical Sciences, Tehran, Iran

**Keywords:** Precipitator, Collection efficiency, Photoelectric, Particulate matter, Emission, Ultraviolet

## Abstract

The importance of studying particulate matter lies in its detrimental impact on human health and the environment. Industrial emissions often carry substantial dust content, necessitating the reduction of their environmental release. This study introduced a laboratory-scale photoelectric precipitator to assess its effectiveness in curbing particle emissions under varying temperature, humidity, and residence time conditions. This device operates in two stages: firstly, it charges particles by exposing copper wire surfaces to ultraviolet rays, generating photoelectrons in the airflow; secondly, it utilizes a positively charged collector surface for absorption and collection. Assessment under different temperature, residence time, and humidity conditions revealed that the system designed for 10 μm diameter particles displayed the highest efficiency. At 150℃, the removal efficiency was 39.55%, rising to 41.34% at 60% humidity and 43.58% with an 18-second residence time. Furthermore, increasing energy consumption from 144 j/l to 720 j/l resulted in a 10.93% efficiency increase, highlighting the correlation between energy input and system efficiency. High particulate matter levels diminish visibility, harm the climate, ecosystems, materials, and contribute to respiratory and cardiovascular ailments. These findings underline the photoelectric precipitator’s potential in mitigating particulate matter’s adverse effects on health and the environment. However, further research is warranted to optimize system design and explore additional parameters’ impact on performance, ensuring its effectiveness in industrial processes to reduce particulate matter emissions.

## Introduction

Industrial processes such as iron foundries produce exhaust gas flow that contains suspended solid particles [1, 2]. These particles can be a by-product of the industrial section or its main product [3]. However, it is necessary to remove these particles from the gas stream as epidemiological research has confirmed the adverse effect of inhalation of airborne particles on human health [4–6]. The particles predominantly hurt the respiratory [7, 8] and cardiovascular systems [9, 10]. The emission control of airborne particles is known as a global challenge, despite developing a variety of control equipment [11, 12]. The results of recent research emphasize the use of new air pollution control technologies based on environmentally friendly mechanisms and energy efficiency in accordance with the goals of sustainable development [13–15]. In recent decades, electrostatic precipitators (ESPs) have been developed, and new methods introduced to increase the particle removal efficiency in the micrometer size range [16–19]. ESPs are one of the suitable equipment for particle removal in industries such as cement [20], solid recovered fuel [21], and coal [22]. Factors affecting the performance of these precipitators are exhaust gas temperature, dew point, particle concentration in the gas, and particle size [23]. ESPs are usually composed of thin wires as discharge electrodes placed between plates with opposite charges (i.e., collecting electrodes). By applying high voltage to these electrodes, a strong electric field is generated, which causes the particles to be charged and then absorbed by the collecting plates, however, their efficiency depends on the particle charge rate [24, 25]. Although ESPs have a low-pressure drop compared to other equipment such as filters or scrubbers [26–28], the energy consumption of this control device is very high and its use and maintenance are very costly for small industries [29]. An electrostatic precipitator (ESP) functions by utilizing electrical energy to charge particles within a gas stream either positively or negatively. These charged particles are subsequently drawn towards collector plates that bear an opposite charge. Depending on the design, the collected particles can be removed from the collector plates in dry form (dry ESPs) or washed away with water (wet ESPs). ESPs are renowned for their high collection efficiencies, often exceeding 99%. Consequently, an ESP typically comprises four main components: gas distribution plates, discharge electrodes, collection surfaces (which can take the form of plates or pipes), and rappers [30]. The photoelectric effect refers to the phenomenon where the emission of electrons or other free carriers occurs when light interacts with a material. Electrons emitted in this manner are commonly referred to as photoelectrons. According to classical electromagnetic theory, this effect can be explained by the transfer of energy from light to an electron. Consequently, a variation in the intensity of light would lead to changes in the kinetic energy of the electrons emitted from the material [31]. In brief, ESP charges the field using high voltage discharge caused by the electromagnetic field, but in the photoelectric mechanism, there is UV radiation without any electromagnetic field. When UV hits the metal cathode (copper wire), it excites and removes electrons from the surface of the metal cathode. Many studies have examined the efficiency of combined ESPs and photoelectric systems [32–34]. Kim W and colleagues used the photoelectric phenomenon (based on UV lamps) to increase the efficiency of ESP in collecting carbon nanoparticles. In this study, with the increase in temperature, the efficiency (ESP) increased, and the reason for this is the increase in the free distance between the particles with the enhancement in temperature or other words, the increase in the mean free motion of the particles, and the distance between the particles is approximately equal to the wavelength of the emitted beam came close to the lamps and the result of this event was the increase of photons impacting the particles and charging the particles more [32]. Matter D and colleagues used KrBr and KrCl Excimer lasers, with a wavelength in the ultraviolet range, to charge submicron carbon particles and metal particles with a size of (60-90-120) nm in the output of diesel engines. According to the reported results, the mechanism of the photoelectric effect depends on the radius of the particles and the size of the surface as well as their work function, so with the increase of the radius of the particles and the size of their surface as well as the decrease of the work function of the particles, this mechanism works more efficiently [35]. Lin Li, Da-Ren Chen carried out the impregnation of aerosols using UV rays (photoelectric mechanism). In this study, airflow containing silver particles in the size range of 7 to 30 nm was passed through the designed reactor with volumetric flow. The result of this study was that the efficiency of collecting silver particles based on the photoionization mechanism was 80% higher than charging them based on the corona mechanism [36]. Zhou et al. aimed to impregnate silver particles (in the range of 10 to 100 nm) with a spherical and mass structure using UV wavelength radiation and focusing on the effect of the particle structure on the quantum field. By measuring the efficiency of charged particles by UV radiation, they concluded that the efficiency of charging for both spherical particles and mass particles is directly proportional to the third power of the particle diameter (D^3) and the efficiency of charging for particles with the spherical structure is more than mass particles [37]. Esther Hontanon and F.Einar Kruis, two mechanisms of charging of nanoparticles (25–5 nm) by UV radiation and charging by radioactive radiation of element ($$ {}_{36}{}^{85}Kr$$) with activity (10 mCi) in high volume flows were compared with each other. The results showed that the use of the photoionization mechanism to charge particles using UV radiation compared to radioactive radiation, with the increase of the volume flow of the airflow, has better performance and efficiency [38].

The photoelectric mechanism is known as an electron quantum phenomenon in which matter, especially metals, emits electrons after absorbing energy from the electromagnetic beams emitted by it. One of the effective factors in the occurrence of photoelectric mechanisms is the frequency of electromagnetic radiation and the work function (WF) of the irradiated metal. The energy of the free electrons on the metal surface increases and the photoelectrons are separated from the surface when ultraviolet photons are irradiated with energy higher than the WF of metal on the metal surface. This mechanism causes electrons to collide with particles by emitting electrons from the metal surface, causing them to charge [39]. Researchers who have used the photoelectric mechanism to charge the particles emit photoelectrons by irradiating light photons to the surface of the particles, which charge the particles while ionizing them. Although in some studies, the photoelectric mechanism has been used as a method to increase the charging of particles in electrostatic precipitators in a consolidated way, so that they can compensate for the amount of charging of particles by reducing the voltage applied to the electrodes and reducing energy consumption by using this mechanism. But if the photoelectric mechanism is used as a method of charging particles (diffusion), it can be classified as an independent method along with other air pollution control methods due to its advantages. In the present study, the photoelectric mechanism was applied to a photoelectric precipitator (PEP) scale depositor using direct irradiation of optical photons of ultraviolet (UV) to the copper metal surface. The study aimed to investigate the collection efficiency of the PEP regardless of the particle’s metallic and non-metallic properties at different temperatures, humidity, and residence time conditions. The results showed that the system can work optimally in terms of energy consumption and provide relatively good efficiency in reducing the emission of particulate pollutants. The system has the advantage of being low cost in construction and maintenance, making it suitable for use in places where access to high voltage is not possible or expensive. Furthermore, connecting several systems in a row is expected to achieve better efficiency, which is suggested to be investigated in future studies. The study suggests that the system may help control particulate matter (PM) in some processes such as iron foundry, welding, grinding, smelting, etc., and thus decrease the exposure risk of workers.

## Materials and methods

### Materials and experimental setup

**Fig. 1:**
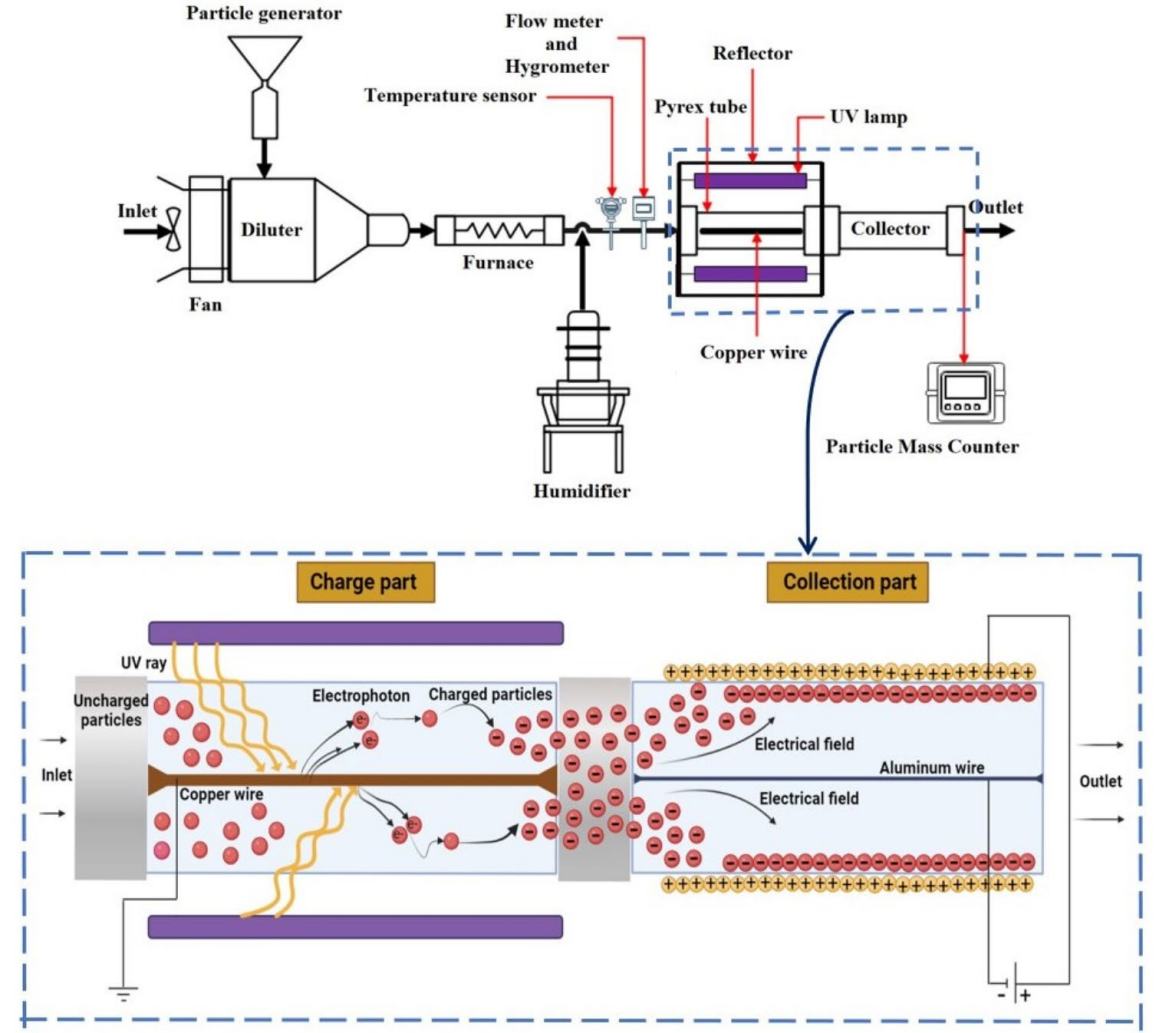
Schematic of experimental setup

Fig. 1, illustrates the test set-up used to evaluate the collection efficiency using a PEP system. The PEP system comprises three main parts, including the particle generation part, particle charging part, and particle collection part. The air blower system used in this study consisted of an axial fan with a diameter of 145 mm and a body of (180 × 180) $$ {mm}^{2}$$, connected to a dimmer that changed the voltage from zero to the source voltage, allowing the volumetric flow of airflow. To investigate the effects of temperature (25℃, 50℃, 100℃, and 150℃) and relative humidity (0%, 20%, 40%, and 60%) on the efficiency of the system in residence time 4 s, 7 s, and 18 s of the humidifier, respectively, an electric oven was used before the dehumidifier, controlled by a hygrometer and a thermal sensor. Temperature and relative humidity were measured at a distance of 3 cm before the particle charge inlet. The electric oven had a length of 35 cm, an inner diameter of 4.5 cm, and a thickness of 3 mm, in which an electric coil was applied to create heating. To access the particle diameter range expected in the study (0.5, 0.7, 1, 2.5, 4, 5, 7, and 10 (µm)), clay particle mass was used. The source of particle generation in the present study consisted of a flat DC armature motor with a voltage of about 5 V, connecting to a gear. The fine particles in the diameter range (1–100 μm) of clay particle mass were poured inside a funnel with a capacity of 200 gr (the opening surface of the funnel was (8 × 8) $$ {cm}^{2}$$, and the outlet with a diameter of 1 cm and was placed perpendicular to the top of the gear. The system, while crushing the particles, directed them to a cylindrical hopper with a conical outlet (the length of the cylinder equal to 5 cm and a conical outlet angle of 15 degrees) with a capacity of 15 gr, and a discharge rate of 0.001 gr/sec, and added to the airflow using the clean air created by the air blower with an emission rate of 0.012 gr/l. Next, the particles are charged by a photoelectric mechanism and collected by the collection part. The particle size distribution emitted into the PEP at a residence time of 7 s and in vitro (SATP^1^) is shown in Fig. 1. The use of clay particle mass can be justified by the high quality of clay components in particles emitted from the chimneys of cement, ceramics, and bricklaying industries, as well as the similarity of quantitative analysis of particulate pollutant components in these industries with materials [40–42].

To prevent thermal failure, UV lamps were placed around a Pyrex tube with a length of 30 cm, an inner diameter of 5 cm, and a thickness of 1.1 mm. The UV rays were able to pass through the Pyrex tube. However, the quartz tubes are preferred. But, in the present study, the Pyrex tube was considered due to some reasons such as: (1) Pyrex demonstrated a significantly higher tolerance to radiation exposure before reaching the non-Ohmic threshold compared to Quartz material. Specifically, Pyrex glass exhibited radiation damage at a relatively high dose rate of 0.25 Gys-1, whereas Quartz deviated from its Ohmic response at approximately 0.07 Gys. (2) Pyrex dosimeters, containing higher impurities, generated photoconductive currents of considerably greater magnitude compared to Quartz, a material characterized by high purity, which produced photoconductive currents of lower magnitude. Therefore, it was inferred that utilizing a base material with a moderate level of impurities would result in photoconductive currents within a more practical range [43]. Considering that Pyrex tube is cheaper than quartz and on the other hand, providing the required tolerance for the current system, Pyrex was used.

To maintain the static balance of the system, the whole set was placed inside a chamber made of Plexiglas with an aluminium inner coating to reflect and focus the optical photons (aluminum was used to cover the reactor to reflect the radiation of UV inside the reactor, as well as to avoid the radiation out of the reactor. It was also, a safety issue). A metal wire made of copper with a diameter of 2 mm and a work function of 4.48–4.94 eV [44, 45] was applied as an electrode inside the Pyrex tube and its coaxial. In other words, one of the important parameters to create the photoelectric effect is the type of cathode or the type of metal that the light rays shine on. This parameter determines the work function. Considering, the work function of copper was close to silver and aluminum (copper metal equal to (4.65 eV), silver metal (4 eV), and aluminum metal (4.19 eV)), the copper was applied as a cathode. This metal wire, which emits electrons by irradiating UV photons on its surface, was connected to an earth wire.

Radiation density is defined as the amount of radiated energy per unit surface area of an object, and it is considered one of the important parameters for explaining the power characteristics of the radiation source. Based on the results of a study, increasing the number of light bulbs increases the efficiency of charging particles [36]. Considering that in the current study, the amount of energy required was calculated to be equal to 24 watts, therefore, to increase the brightness of the radiation, two UV lamps with a power of 12 W and a wavelength in the range of 254.7 nm were used Two. The important advantage of the designed photoelectric reactor based on the UV lamp is its applicability to collect particles, regardless of their physical and chemical nature. The collection part was made of a steel tube with a length of 30.5 cm, an inner diameter of 4.9 cm, and a thickness of 3 mm. The aluminium wire with a diameter of 3 mm was placed along with the collector, and the gap distance between the aluminium wire and the collector plate was 2.35 cm.

**Fig. 2:**
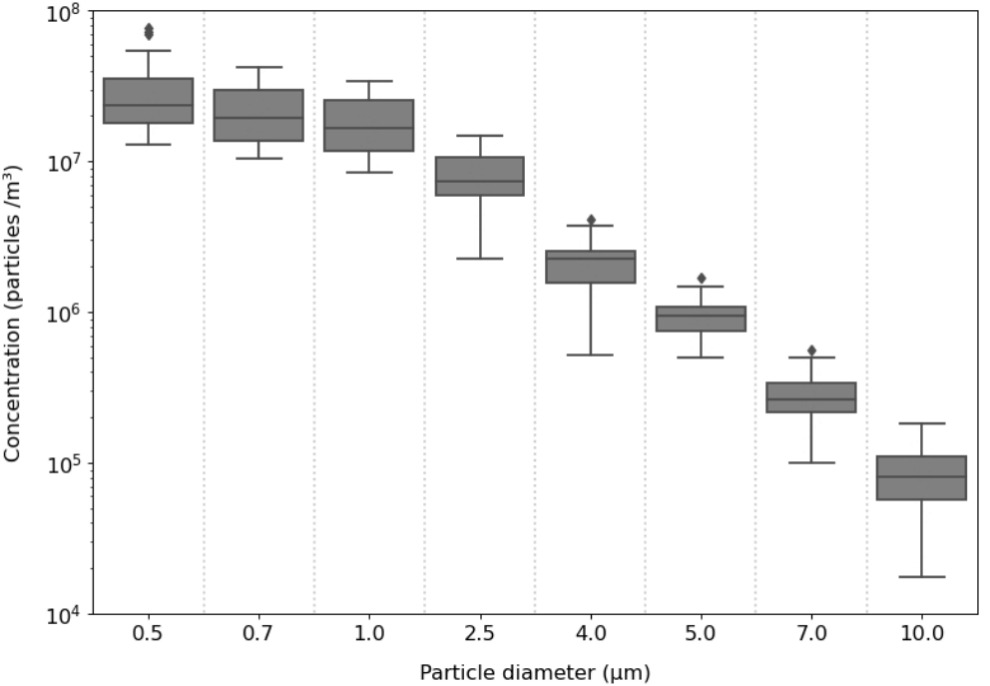
Distribution of particles produced by particle generator

The efficiency of the PEP was evaluated by measuring the number of particles in eight size ranges: 0.5, 0.7, 1, 2.5, 4, 5, 7, and 10 (µm) Fig. 2. The PEP’s collection efficiency was measured in each test design, considering isokinetic, using the Particle Mass Counter device model TES-5200 made by TES & PROVA Taiwan with 5% accuracy.

### Calculations

The electric field inside the collector was calculated using Eq. 1.

1 $$ E(v/m)= \frac{V\left(volt\right)}{D\left(distance\right)}$$

Where, E is electric field, V is applied voltage between Al wire and collector plate, and D is the gap distance between Al wire and collector plate.

To evaluate the optimal conditions of energy consumption in PEP, the energy density or energy consumed per unit of Pyrex tube volume (particle charging part) was calculated using the following Eq. 1 [46, 47].

2 $$ SIE(j/l) =\frac{P \left(W\right)}{Q (l/s)}$$

Where, P is discharge power, Q is gas flow rate, and SIE is Specific Input Energy for the particulate matter collection.

Collection efficiency, η, can be calculated as Eq. 3.

3 $$ \eta =\left(1- \frac{{N}_{outlet}}{{N}_{inlet}}\right) \times 100\text{\%} $$

Where, $$ {N}_{inlet} $$and $$ {N}_{outlet}$$ represent the number of particles at the inlet and outlet of the PEP.

## Results and discussion

The present study aimed to analyze the effect of temperature, residence time, and humidity on the rate of particle removal from airflow for different ranges of particle diameters. The study reported optimal particle removal conditions for all particle size ranges.

### Effect of temperature

**Fig. 3:**
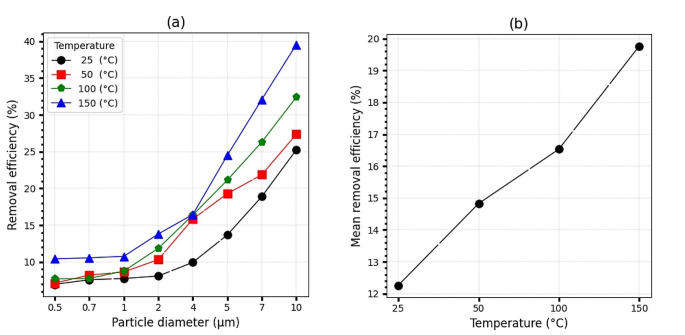
(**a**): PEP removal efficiency in terms of particle diameter in different temperature conditions. (**b**): PEP mean removal efficiency in different temperature conditions

Experiments were conducted to investigate the effect of airflow temperature on the particle removal efficiency by the PEP system in terms of particle diameter. The results of the study are presented in Fig. 3a.

The study found that the highest system efficiency of 39.55% was observed at 150 ℃ for particles with a diameter of 10 μm, as shown in Fig. 3b. The system efficiency increased with increasing temperature and was higher for particles with a diameter of 10 μm, as shown in Fig. 3a. On average, for all particle ranges, system efficiencies at 25℃, 50℃, 100℃, and 150 ℃ were obtained as 12.26%, 14.82%, 16.53%, and 19.76%, respectively.

Kim et al. obtained a similar result, with increasing temperature from 694 to 839°C, the efficiency of the photoelectric-ESP integrated system for particles with a diameter of 18.4 nm increased by 28.5% [32]. Additionally, Zheng et al. discovered that when the temperature was increased from 300 to 363°K, the rate of particle charge of 0.73 μm Increaced by 30% [48]. Based on the results of the present study, it was found that increasing the temperature from 25℃ to 150 ℃ resulted in an average increase of 7.50% in the system efficiency for particles with a diameter of 10 μm. The increase in efficiency due to the increase in the temperature of the airflow can be attributed to two factors: the decrease in the volume density of the airflow and the increase in the mobility of ions. The decrease in volume density of the airflow results in an increase in the current density, which subsequently causes an increase in the charge rate of the particles [49, 50]. With increasing temperature, system efficiency has improved. However, on average, all four temperature ranges studied are close to each other.

### Effect of relative humidity

**Fig. 4:**
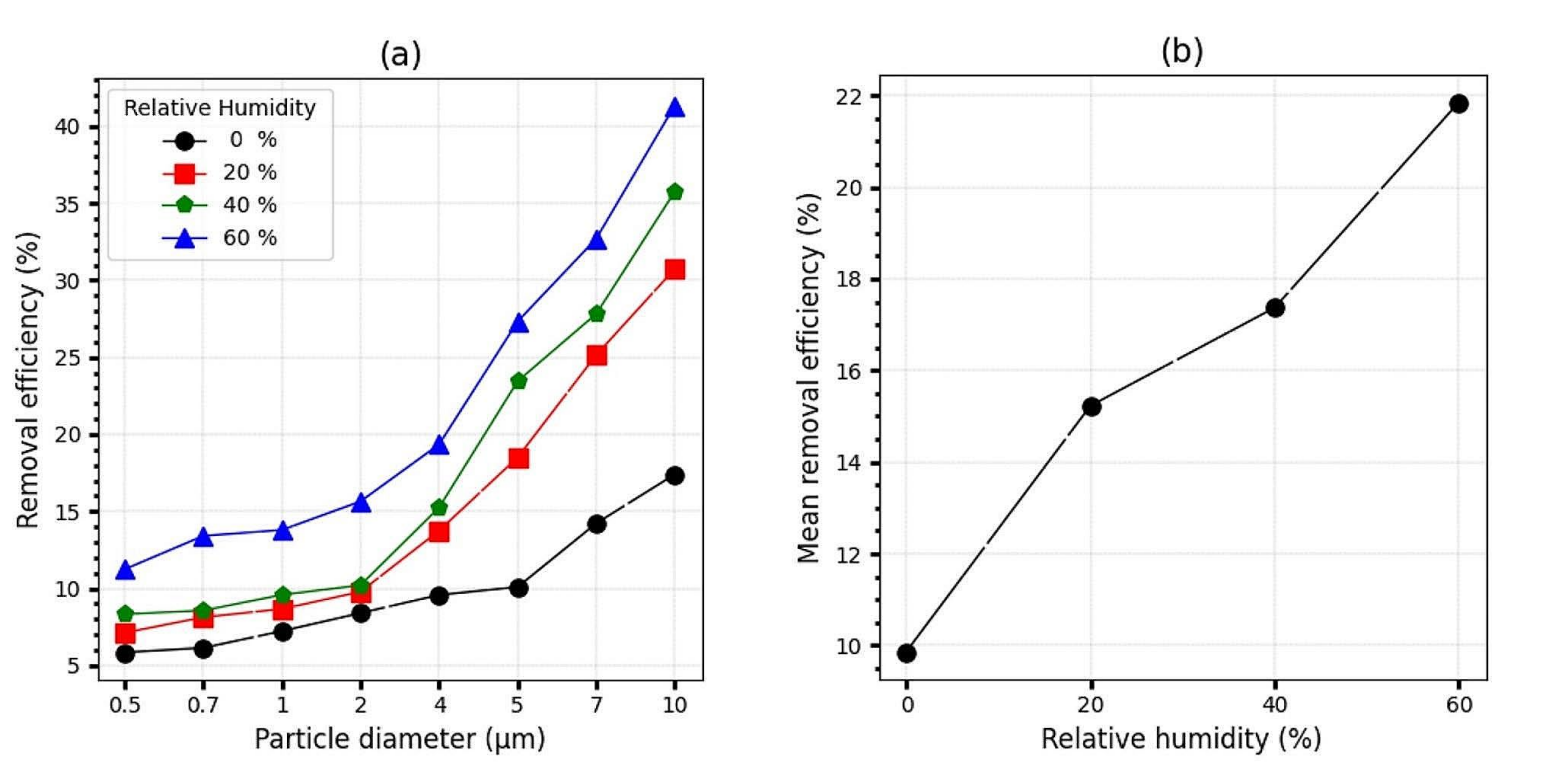
(**a**): PEP collection efficiency in terms of particle diameter in different relative humidity conditions. (**b**): PEP collection efficiency in different relative humidity conditions

According to the results shown in Fig. 4, the effect of four levels of relative humidity (10%, 20%, 40%, and 60%) on the mean removal efficiency was demonstrated. In general, the relative humidity of the airflow in all particle purification equipment is crucial because it is in the form of water droplets in the size of microns. Two reasons are suggested. Firstly, water droplets are in the form of particles, and increasing the number of particles, regardless of their nature, causes them to have the ability to trap particles smaller than their size and may exist in the system as larger particles [51]. Secondly, the combined mass of the particle increases with the water droplets, and the amount of sediment due to its weight is debatable [52]. The presence of relative humidity in the airflow has been found to increase the ionization coefficient. This increase intensifies the ionization process of neutral molecules, leading to an increase in the density of ions. As a result, the number of negative ions that move to the weaker parts of the electric field increases, and the possibility of collision of ions with particles also increases [48]. When particles absorb water vapor, the vapor condenses on their surface, increasing the water content of the particles. The polarizability of wet particles in the presence of an electric field is higher than that of dry particles, which increases the probability of collision between ions and particles, subsequently increasing the charge of particles [53]. The system efficiency of a particle system is affected by various factors such as particle diameter and humidity levels. For particles with a diameter of 10 μm, the system efficiency was highest at 41.34%, while the system efficiency for all particle ranges was on average 9.86%, 15.22%, 17.36%, and 21.84% at humidity levels of 0%, 20%, 40%, and 60%, respectively. Additionally, the system efficiency increased by 11.98% across the entire particle diameter range as relative humidity increased from 0 to 60%. The effect of relative humidity on the efficiency of an ESP has been investigated in several studies [48, 54]. Nouri et al., found that increasing the relative humidity during ESP operation will increase the collection efficiency [44], and relative humidity level has a significant impact on discharge current [55]. It is along with the results of the study.

### Effect of the residence time

**Fig. 5:**
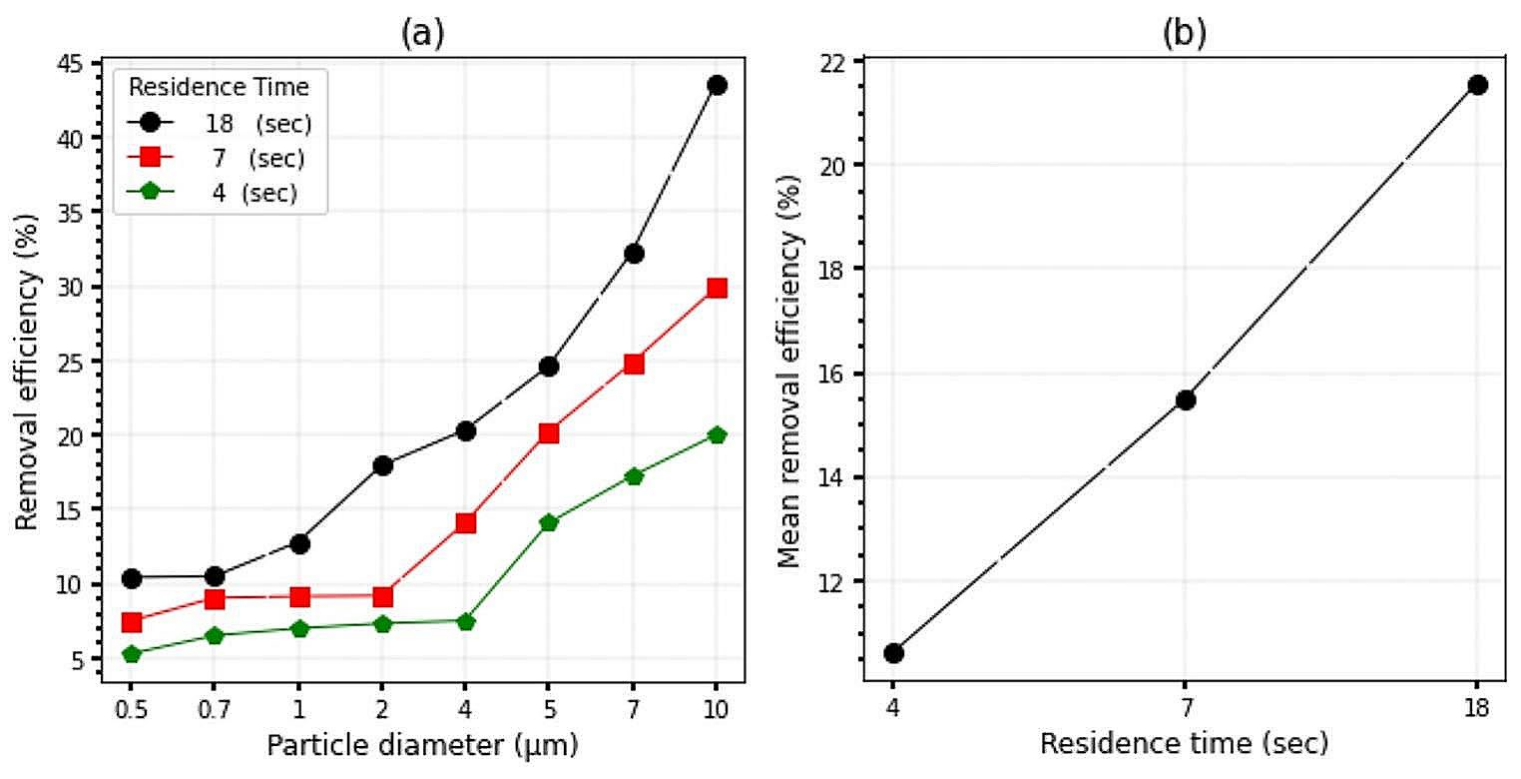
(**a**): PEP collection efficiency in terms of particle diameter in different residence time conditions. (**b**): PEP collection efficiency in different residence time conditions

In the present study, three different levels of airflow residence time were tested and the efficiency of particle removal for particles of different diameters was measured. The experimental results showed that the highest efficiency of 43.58% was achieved for particles with a diameter of 10 μm at a residence time of 18 s. Conversely, the lowest efficiency of 5.30% was obtained for particles with a diameter of 0.5 μm at a residence time of 4 s. These results are presented in Fig. 5a.

The relationship between the residence time of particles inside a system and the charging of those particles has been studied in various research articles [56, 57]. The longer the particles stay, the more likely they are to be charged by electrons hitting them. Figure 5b shows that as airflow increases or the time of presence of particles inside the system decreases, the average efficiency of the system decreases significantly. This trend has been observed in previous studies [48, 58]. The efficiency of the system decreases by 10.93% on average and for the whole range of particle diameters when the residence time is increased from 4 s to 18 s, indicating that the system is more efficient for lower flow rates. Figure 5b also shows that for the particle diameter range studied, the average system efficiencies for residence time 18 s, 7 s, and 4 s were 21.56%, 29.86%, and 19.99%, respectively.

### Effect of the residence time on the specific energy input

**Fig. 6:**
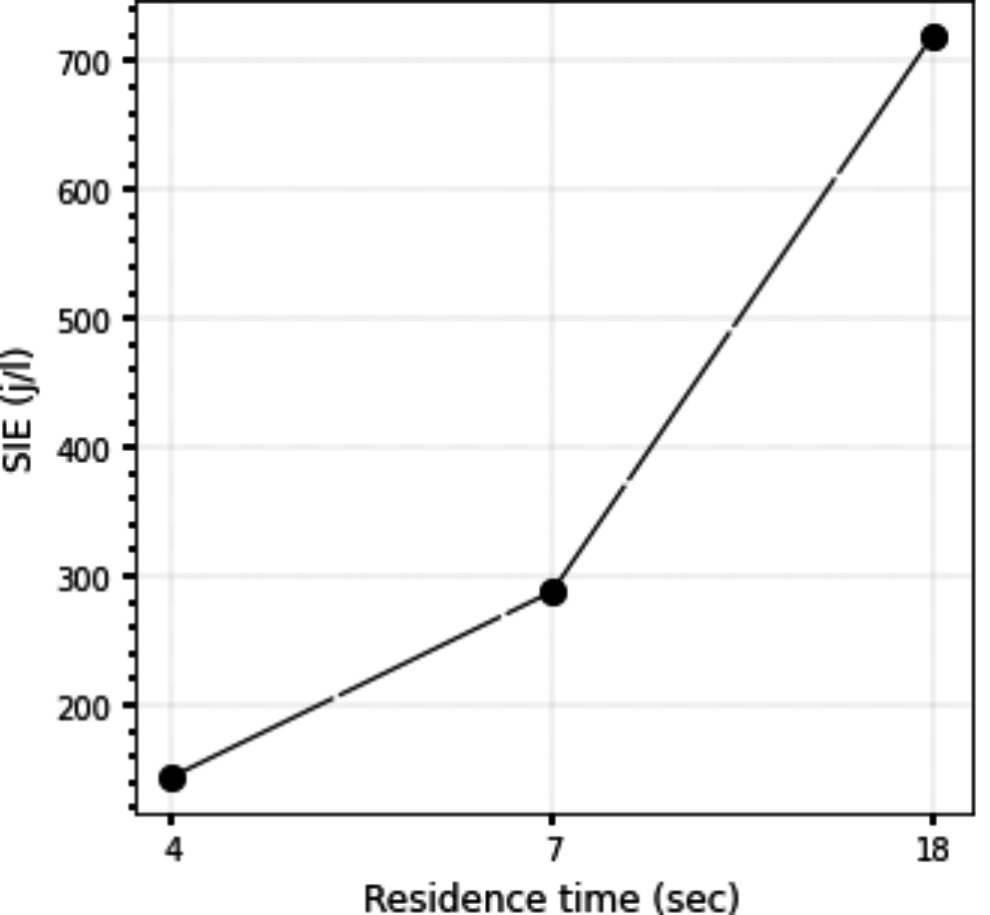
Energy consumption of the system per particle’s residence time

In the present study, an applied voltage of 220 V and an electric field of 9361 v/m were utilized. To achieve the aim of the study, which was to minimize energy consumption in PEP, the SIE was calculated by determining the time the particles remained in the charging part of the particles. The results were presented in Fig. 6.

The energy consumption of PEP at different residence times. The energy consumption values are 720 j/l, 288 j/l, and 144 j/l for residence times of 18 s, 7 s, and 4 s, respectively, as shown in Fig. 6. It is observed from Fig. 6 that the energy consumption of PEP is at its lowest value of 144 j/l in the residence time of 4 s. As the residence time decreases, there is a significant reduction in the value of SIE, indicating a decrease in energy consumption. C. Anderlohr et al. found that the amount of SIE increases significantly as the residence time of particles in the system increases [59]. Additionally, the efficiency of the system increases with the increase in residence time, as shown in Fig. 5. Specifically, the PEP efficiency increases by 10.93% as the energy consumption rises from 144 j/l to 720 j/l. Based on economic considerations regarding energy consumption and expected PEP efficiency, optimal conditions for the application of this system were chosen. By studying the optimal performance conditions of the designed system, the optimal performance efficiency was obtained equally at 30% at the temperature of 150 ^o^C Fig. 3a, the resistance time of 7s Fig. 5a, the relative humidity of 20% Fig. 4a, and SIE of 300 ($\frac{j}{\text{l}})$ for particles with a diameter of 10 µ with the use of copper cathode.

## Conclusion

In this study, the effectiveness of a PEP in removing suspended solid particles from industrial exhaust streams was investigated. The study examined the impact of temperature, relative humidity, residence time, and SIE on the PEP system’s efficacy, providing crucial insights into air pollution control strategies. The study found that increasing the airflow temperature improved the PEP system’s efficiency, with a peak efficiency of 39.55% observed at 150 °C for 10 μm particles. The efficiency increase was consistent across particle sizes, with an average increase of 7.50% as the temperature increased from 25 °C to 150 °C. Relative humidity was also found to have a significant impact on the system’s efficiency, with higher humidity levels leading to superior efficiency. A 60% relative humidity level resulted in an average efficiency of 21.84% across all particle sizes, representing an 11.98% increase compared to 0% relative humidity. The study revealed that extended residence times within the PEP system were linked to heightened particle removal efficiency, with the peak efficiency of 43.58% recorded at an 18-second residence time for 10 μm particles. Conversely, the lowest efficiency of 5.30% was observed at a 4-second residence time for 0.5 μm particles. The calculation of SIE using a 220 V applied voltage and a 9361 V/m electric field unveiled that a 4-second residence time minimized energy consumption at 144 J/l. Decreasing residence time led to reduced SIE, signifying lower energy utilization. Furthermore, extended residence times resulted in a 10.93% increase in system efficiency as energy consumption rose from 144 J/l to 720 J/l. Development of the designed system at the field scale may be an efficient system for the treatment of emitted pollutants in some industries such as gypsum processing during Plasterboard and Plaster manufacturing, iron ore sintering process, and so on. These findings provide valuable knowledge for optimizing environmentally friendly and economically efficient operational parameters for the PEP system, bolstering its role in mitigating industrial air pollution.

## Footnotes

1 Standard Ambient Temperature and Pressure (T = 25 (°C), *P* = 101.3 (Kpa)).
